# The Role of Percentage Signal Recovery (PSR) in MRI Perfusion for the Diagnosis of Primary Central Nervous System Lymphoma

**DOI:** 10.7759/cureus.88495

**Published:** 2025-07-22

**Authors:** Eliezer Villanueva-Castro, Marco Antonio Muñuzuri-Camacho, Luis A Rodríguez-Hernández, Bernardo Cacho-Díaz, Vicente Guerrero-Juarez, Rebeca Hernández Reséndiz, Tomas Moncada-Habib, Rodolfo Villalobos-Díaz, José Guillermo Flores-Vázquez, Domingo J Coutinho-Thomas, Jorge Omar Garcia-Gutierrez, Guillermo A Gutierrez-Aceves, Alberto González-Aguilar

**Affiliations:** 1 Neurosurgery, University of Arkansas for Medical Sciences, Little Rock, USA; 2 Neurosurgery, Instituto Nacional de Neurología y Neurocirugía Manuel Velasco Suárez, Mexico City, MEX; 3 Neuro-oncology, Instituto Nacional de Cancerología, Mexico City, MEX; 4 Neurology, Instituto Nacional de Neurología y Neurocirugía Manuel Velasco Suárez, Mexico City, MEX; 5 Pediatric Neurology, Hospital Angeles Universidad, Mexico City, MEX; 6 Demyelinating Diseases, Instituto Nacional de Neurología y Neurocirugía Manuel Velasco Suárez, Mexico City, MEX; 7 Radiology, Angeles del Pedregal Hospital, Mexico City, MEX; 8 Radioneurosurgery, Instituto Nacional de Neurología y Neurocirugía Manuel Velasco Suárez, Mexico City, MEX; 9 Neuro-oncology, Centro Oncologico Internacional, Mexico City, MEX

**Keywords:** cerebral blood volume, diagnostic accuracy, mri perfusion, neuroimaging biomarkers, percentage signal recovery, primary cns lymphoma

## Abstract

Introduction

Primary central nervous system lymphoma (PCNSL) is a rare type of malignancy confined to the central nervous system. While histopathological analysis remains the gold standard for diagnosis, it is invasive and not always feasible. Percentage signal recovery (PSR) derived from perfusion-weighted magnetic resonance imaging (MRI) has emerged as a promising non-invasive biomarker for PCNSL.

Objective

This study aims to evaluate the diagnostic accuracy of PSR and relative cerebral blood volume (rCBV) in distinguishing PCNSL from other CNS mass lesions using perfusion MRI.

Methods

We conducted a retrospective study using the neuro-oncology database of a national referral center in Mexico. Patients with histopathological confirmation and available perfusion MRI were included in the analysis.

Results

From a total of 16,198 patients, 700 met the inclusion criteria and were classified as follows: PCNSL (n = 86), high-grade gliomas (HGGs; n = 435), metastases (n = 80), neuroinfections (n = 80), and pseudotumoral demyelinating disorders (PDDs) (n = 19).

Mean rCBV for PCNSL was 1.1, HGGs 3.9, metastases 3.0, neuroinfections 0.5, and PDDs 0.9, while mean PSR for PCNSL was 176%, HGGs 92%, metastases 81%, neuroinfections 95%, and PDDs 91%, with a statistically significant difference for PCNSL (p ≤ 0.0003).

Receiver operating characteristic (ROC) curve analysis showed that PSR was the only parameter with robust diagnostic performance. A PSR threshold of >110 yielded 98% sensitivity (95% CI: 90.7-100) and 99% specificity (95% CI: 91.3-100) for the diagnosis of PCNSL.

Conclusion

PSR demonstrated high diagnostic accuracy in differentiating PCNSL from other enhancing brain lesions and may be particularly valuable in patients with atypical presentations, multiple comorbidities, or nondiagnostic or contraindicated biopsies. PSR is a promising non-invasive imaging biomarker that warrants further prospective validation.

## Introduction

Primary central nervous system lymphoma (PCNSL) has been reported with increasing frequency in recent years, accounting for approximately 1%-6% of all central nervous system (CNS) malignancies [1,2]. Neuroimaging commonly reveals one or more lesions, often located in the periventricular region, with variable contrast-enhancement patterns. Typical magnetic resonance imaging (MRI) characteristics of PCNSL include hypo- or iso-intensity on T1-weighted images (T1WI) and iso- or hyperintensity on T2-weighted images (T2WI) [3-7].

Although MRI findings can be highly suggestive, histopathological confirmation remains essential [8]. Stereotactic biopsy plays a key role in the diagnosis of PCNSL, given the typical size, location, and depth of the lesions, and is not associated with worsened survival outcomes [9]. However, it presents several limitations: adequate tissue sampling is not always achievable, and many patients receive corticosteroids or cytotoxic drugs prior to biopsy, leading to partial or complete lesion regression and complicating diagnostic accuracy, particularly for lymphomas [10-12].

Moreover, MRI features of PCNSL often overlap with those of other intracranial pathologies, including high-grade gliomas (HGGs), meningiomas, metastases, neuroinfections, and pseudotumoral demyelinating disorders (PDDs), which further challenges the diagnostic process [13-15]. In recent years, advanced neuroimaging techniques such as diffusion tensor imaging, MR spectroscopy, positron emission tomography with fluorodeoxyglucose (FDG-PET), and perfusion-weighted MRI have been proposed as complementary tools to differentiate PCNSL from its mimics.

Among these, perfusion MRI parameters, specifically relative cerebral blood volume (rCBV) and percentage signal recovery (PSR), have shown potential utility in the evaluation of brain lymphomas [16-18]. Given the diagnostic challenges and the need for noninvasive biomarkers, our study aims to evaluate the role of PSR and rCBV derived from perfusion MRI in distinguishing PCNSL from other CNS lesions, using histopathology as the reference standard.

## Materials and methods

Patient selection

**Figure 1:**
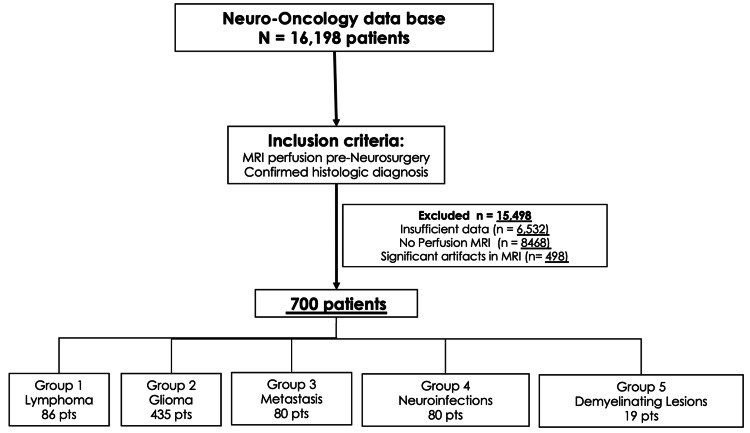
Flowchart of patient selection criteria and inclusion process for the study cohort Flow diagram illustrating the screening and selection process of patients from the Neuro-Oncology database, including inclusion and exclusion criteria applied to obtain the final sample.

A retrospective search was conducted in the Neuro-Oncology database of the National Institute of Neurology and Neurosurgery in Mexico to identify patients with primary and metastatic brain tumors, as well as neuroinfections, who underwent MRI perfusion studies prior to any intervention. Only patients with histopathological confirmation and available perfusion MRI for re-analysis were included (Figure 1).

Data collection

From a total database of 16,198 patients, 700 met the inclusion criteria and were analyzed retrospectively (Figure 1).

Imaging protocol

All images were acquired on a 3-Tesla MRI scanner (General Electric Medical Systems) using the following conventional sequences: sagittal T1 FLAIR (TR 2000 ms, TE 21.2 ms, TI 750 ms, Nex 1); axial FLAIR diffusion-weighted imaging (TR 11002 ms, TE 133.5 ms, Nex 2); axial T1-weighted images (TR 440 ms, TE 16 ms, Nex 3) before and after intravenous administration of gadolinium contrast (SPGR with inversion recovery, TR 7.4 ms, TE 2.4 ms, TI 450 ms, Nex 0.81); and axial T2-weighted images (TR 4400 ms, TE 117.9 ms, Nex 3) after gadolinium administration.

Perfusion-weighted imaging (PWI) was performed using T2*-weighted gradient echo echo-planar imaging (GRE-EPI) sequences, acquiring 10 contiguous 5-mm slices without gaps, covering the lesion identified on conventional sequences. Gadopentetate dimeglumine was administered at a standard dose of 0.1 mmol/kg as a manual bolus, followed by 20 mL of saline flush. T2*-weighted images were obtained every two seconds during the first pass of the contrast agent to achieve high temporal resolution.

Post-processing was performed using FuncTool 2 software on a GE Healthcare workstation. Regions of interest (ROIs) of 30-40 mm² were manually placed on the enhancing tumor areas on T1-weighted images to determine maximum, minimum, and mean PSRs. Non-enhancing necrotic areas were excluded. For normalization, ROIs of 30-50 mm² were also placed on contralateral normal-appearing white matter to calculate relative values. Only lesions with a minimum diameter greater than 10 mm were included to ensure valid ROI placement. No motion correction or automated segmentation tools were applied during post-processing.

Percentage signal recovery (PSR) calculation

**Figure 2:**
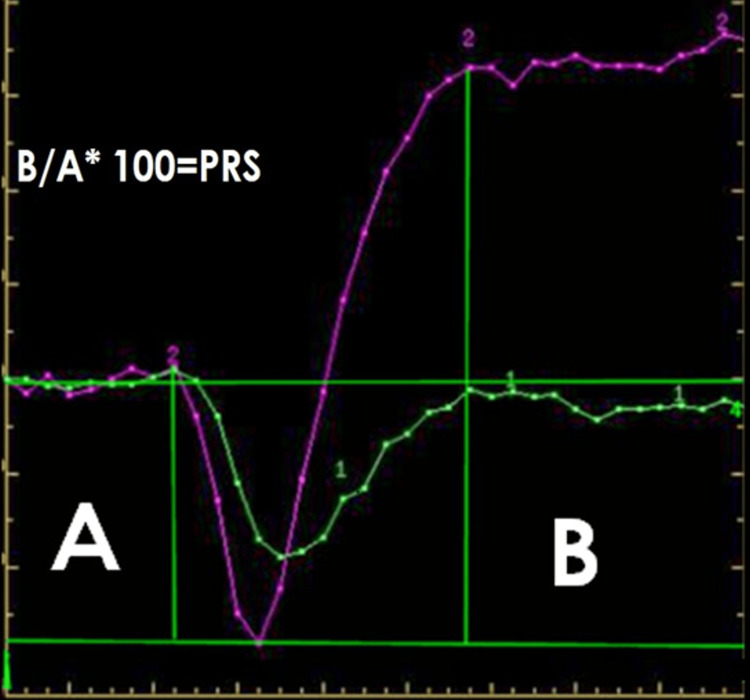
Calculation of percentage signal recovery (PSR) T2*-weighted signal intensity was measured before (A) and after (B) contrast administration. The PSR was calculated using the following formula: PSR=(B/A)×100 where A represents the pre-contrast baseline signal intensity and B corresponds to the post-contrast recovery signal intensity. The green line depicts the signal drop following contrast injection, while the purple line shows the subsequent recovery over time.

PSR was calculated using Cha’s formula, where PSR equals 100% times the difference between S1 (post-contrast T2*-weighted signal intensity) and Smin (minimum signal intensity during the dynamic curve) divided by the difference between S0 (pre-contrast signal intensity) and Smin (Figure 2).

Three independent reviewers blinded to pathological diagnosis performed the image analysis and PSR measurements.

Statistical analysis

Normality of continuous variables was assessed using the Shapiro-Wilk test, which indicated non-normal distribution. Therefore, non-parametric tests were applied: the Mann-Whitney U test for comparisons between two groups and the Kruskal-Wallis test for multiple groups.

Median PSR values and interquartile ranges were calculated for each group. Diagnostic accuracy of PSR for differentiating primary CNS lymphoma from other brain lesions was evaluated using ROC curves derived from logistic regression models.

Optimal cutoff values were selected based on the highest sensitivity and lowest false-positive rate.

In addition to sensitivity and specificity, positive and negative likelihood ratios (LR+ and LR-) were calculated for each cutoff point to complement clinical interpretation of diagnostic performance.

All analyses were performed with MedCalc version 15.8. A p-value < 0.05 was considered statistically significant.

## Results

A total of 700 patients met the inclusion criteria and were classified into five groups: PCNSL (n = 86, 12.3%); HGGs (n = 435, 62.1%); brain metastases (n = 80, 11.4%); neuroinfections, including tuberculoma, cerebral abscess, toxoplasmosis, neurosyphilis, neurocysticercosis, and encephalitis (n = 80, 11.4%); and PDDs, including pseudotumoral lesions and acute disseminated encephalomyelitis (n = 19, 2.7%).

Sociodemographic characteristics by group are summarized in Table 1.

**Table 1:** Sociodemographic characteristics and PSR values by diagnostic group (n = 700) PSR: percentage signal recovery, PCNSL: primary central nervous system lymphoma, HGG: high-grade glioma. PSR values are expressed as medians with interquartile ranges (IQR). Sex is reported as n (%). Age is reported as the mean value for each group. The p-value for PSR comparison was obtained using the Kruskal–Wallis test (H = Kruskal–Wallis statistic). A p-value < 0.05 was considered statistically significant.

Variable	PCNSL (n = 86)	HGG (n = 435)	Metastasis (n = 80)	Neuroinfections (n = 80)	Demyelinating disease (n = 19)
PSR (median, %)	176 (IQR 102.7–387.5)	92.1 (34-159.3)	81.4 (78-100)	95.3 (80-102.9)	98.6 (83.3-107.6)
Female, n (%)	34 (39.5%)	161 (37.0%)	28 (35.0%)	33 (41.3%)	15 (78.9%)
Male, n (%)	52 (60.5%)	274 (63.0%)	52 (65.0%)	47 (58.8%)	4 (21.1%)
Age (mean, years)	52.3	42.4	54.5	44.6	35.8

None of the patients were HIV-positive.

PSR differences between PCNSL and other pathologies

The median PSR (interquartile range) for each group was as follows: PCNSL, 176 (102.7-387.5); HGGs, 92.1 (34-159.3); metastases, 81.4 (78-100); neuroinfections, 95.3 (80-102.9); and PDDs, 98.6 (83.3-107.6).

**Figure 3:**
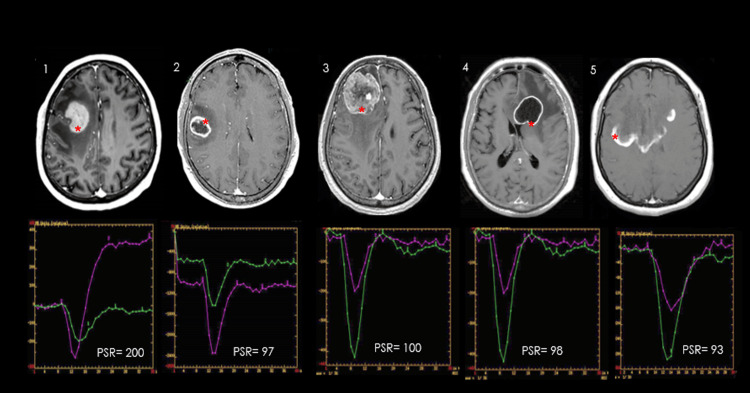
Comparison of percentage signal recovery (PSR) values across different CNS pathologies Post-contrast axial T1-weighted MR images alongside corresponding perfusion curves from five central nervous system (CNS) pathologies: primary CNS lymphoma (PCNSL), glioblastoma (GBM), breast cancer metastasis, cerebral tuberculoma, and pseudotumoral demyelinating lesions. In each case, a red asterisk (*) indicates the manually placed tumor region of interest (ROI) within the enhancing component of the lesion. Corresponding perfusion curves show signal intensity over time for the tumor (purple line) and the contralateral control region (green line), along with the calculated PSR value.

Using the Mann-Whitney U test, PSR values in the PCNSL group were significantly higher compared to each of the other groups (p < 0.0001 for all comparisons). These differences are visually illustrated in Figure 3, which displays post-contrast axial T1-weighted MR images and corresponding perfusion curves for representative cases from each group. Notably, a markedly elevated PSR value (200) is observed exclusively in the PCNSL case, reinforcing its potential as a distinguishing imaging biomarker.

Diagnostic performance of PSR for PCNSL

ROC curve analysis identified an optimal PSR cutoff of greater than 112 to differentiate PCNSL from other etiologies, with a sensitivity of 93.9% (95% CI: 87.8-97.5) and a specificity of 99.5% (95% CI: 98.2-99.9).

**Figure 4:**
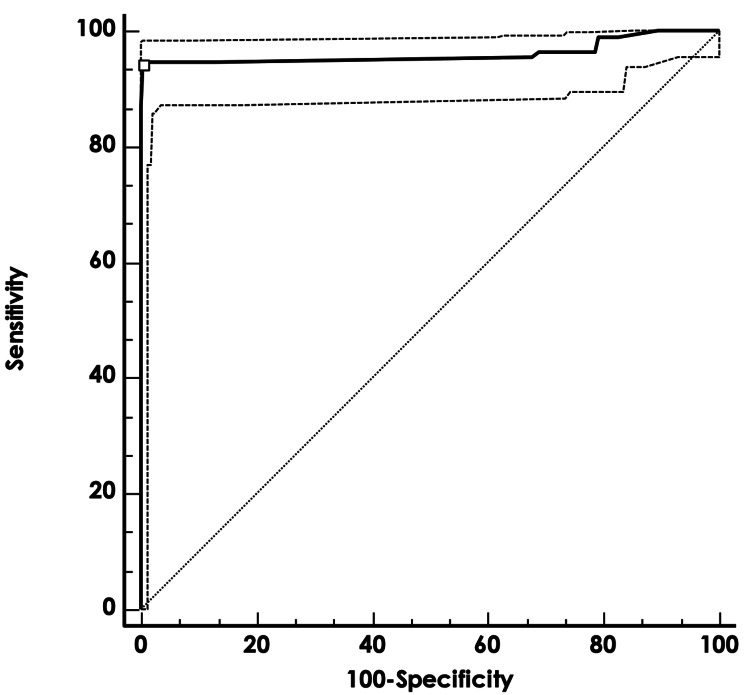
Receiver operating characteristic (ROC) curve for percentage signal recovery (PSR) in diagnosing primary CNS lymphoma (PCNSL) ROC curve illustrating the diagnostic performance of PSR to differentiate PCNSL from other CNS lesions. The area under the curve (AUC), sensitivity, and specificity values at different PSR thresholds are shown.

Higher PSR thresholds yielded even greater specificity. Full diagnostic metrics, including positive and negative likelihood ratios (LR+ and LR-), are presented in Table 2 and illustrated in Figure 4.

**Table 2:** Diagnostic performance of percentage signal recovery (PSR) thresholds for identifying primary central nervous system lymphoma (PCNSL) This table shows the sensitivity, specificity, positive likelihood ratio (LR+), and negative likelihood ratio (LR-) for various PSR cutoff points used to differentiate PCNSL from other CNS lesions. LR+ and LR- were calculated as follows: LR+ = Sensitivity / (1 - Specificity) LR- = (1 - Sensitivity) / Specificity An LR+ greater than 10 is considered strong evidence to rule in the diagnosis, while an LR- less than 0.1 supports ruling it out, aiding clinical decision-making. Infinite (∞) values occur when specificity is 100%, indicating perfect rule-in ability at that cutoff. The asterisk (*) and bold font indicate the optimal cutoff point (>112) selected based on the best balance between sensitivity and specificity.

PSR criterion	Sensitivity (%)	Specificity (%)	LR+	LR–
≥57	100.00	0.00	∞	0.00
>89	100.00	10.46	1.12	0.00
>90	99.12	17.27	1.20	0.05
>94	99.12	20.68	1.25	0.05
>95	96.49	21.41	1.23	0.16
>96	96.49	31.14	1.40	0.11
>97	95.61	32.12	1.41	0.14
>98	94.74	87.10	7.27	0.06
>103	94.74	98.54	63.81	0.05
>105	93.86	99.27	126.42	0.06
>108	93.86	99.27	126.42	0.06
>112 *	93.86	99.51	192.35	0.06
>115	86.84	100.00	∞	0.13
>320	0.00	100.00	0.00	1.00

These findings support the role of PSR as a non-invasive imaging biomarker to aid in the differentiation of PCNSL from other contrast-enhancing brain lesions.

## Discussion

PCNSL presents a challenge for physicians and researchers due to its preoperative diagnostic difficulty, which is highly clinically relevant since treatment strategies differ substantially and prognosis is markedly distinct [19]. MRI with perfusion sequences is a technique that provides information related to capillary and microvascular permeability [20]. PCNSL exhibits a characteristic signal intensity curve with a significant increase above baseline, caused by extensive leakage of contrast into the interstitial space [21]. ​​​​​Hartmann et al. [15] and Mangla et al. [20] described percentage signal recovery (PSR) as a useful tool in distinguishing lymphoma from other brain lesions.

The normal recovery signal represents the intensity measured at the end of the first pass of contrast. In PCNSL, there is an initial drop in signal intensity followed by an “overshoot” above baseline [22]. This overshoot phenomenon has been widely described as suggestive of lymphoma. However, the optimal diagnostic cutoff remains unclear. Mangla et al. reported that a PSR > 94 had a sensitivity of 100% and a specificity of 69% [20]. In our study, a higher cutoff of >110 yielded a sensitivity of 98% and a specificity of 99%, which is higher than the average reported by Mangla et al. [20].

The high PSR observed in PCNSL can be attributed to the formation of multiple dense perivascular layers and perivascular widening, leading to increased permeability and endothelial disruption, allowing contrast to remain in the tissue longer [23].

For gliomas, several studies have used MRI perfusion to assess rCBV, but few have examined PSR directly. In our cohort, we identified a PSR cutoff > 92.2, which showed moderate sensitivity and specificity for distinguishing HGGs. Previous studies have reported lower cutoffs, such as 78.22% and 80.95% [18,24]. These differences may be explained by the vascular characteristics of gliomas, which feature abundant vessels and simple vascular hyperplasia. Contrast leaks through these multiple communications and tends to remain below the baseline [25].

In the case of metastases, we observed a cutoff < 100, which resulted in high sensitivity (100%) but low specificity (24.62%). Other studies have reported even lower PSR values, such as 53.46% and 62.5% [18,24]. It is challenging to compare across studies, as brain metastases may share the vascular properties of their primary tumors. While all metastases exhibit vascular fenestrations, renal cell carcinoma and melanoma are notably more hypervascular.

To our knowledge, there are no previous reports comparing demyelinating and infectious diseases as differential diagnoses for PCNSL using PSR. We considered it essential to include these conditions in our analysis to establish their perfusion profiles. The high PSR observed in some of these lesions could be explained by inflammatory changes. Although demyelinating lesions do not typically demonstrate increased vascularity on angiography or histopathology, recent studies describe the presence of normal or inflamed vessels and mild inflammatory angiogenesis. This mechanism may also apply to neuroinfections and help explain the elevated PSR values in these groups [23,25].

In summary, the differences observed in perfusion MRI across pathologies are consistent with their distinct vascular and permeability characteristics. Our findings support the use of PSR as a non-invasive, quantitative imaging biomarker to aid in the differentiation of PCNSL from other contrast-enhancing brain lesions.

Limitations

This study has several limitations. Its retrospective nature may introduce selection bias, and no formal inter-observer variability analysis, such as the intraclass correlation coefficient (ICC), was conducted for PSR measurements, limiting the assessment of reproducibility. Imaging was performed on a single MRI scanner, which may affect generalizability to other platforms. Additionally, potential effects of pre-imaging treatments, such as corticosteroids, were not controlled. Lastly, histological heterogeneity among metastases and infections could influence PSR values. Future multicenter studies with standardized protocols are needed to validate and expand upon these findings.

## Conclusions

Our study suggests that perfusion-weighted MRI, specifically PSR, may be a valuable noninvasive tool for differentiating PCNSL from other contrast-enhancing brain lesions. Given its high sensitivity and specificity in our cohort, PSR could be particularly helpful in patients with multiple comorbidities, high surgical risk, or inconclusive biopsy results. However, these findings are based on a retrospective, single-center analysis and should be interpreted with caution. Prospective multicenter studies are warranted to validate the clinical utility of PSR before its widespread adoption in diagnostic workflows.
